# Flow cytometry and K-mer analysis estimates of the genome sizes of *Bemisia tabaci* B and Q (Hemiptera: Aleyrodidae)

**DOI:** 10.3389/fphys.2015.00144

**Published:** 2015-05-19

**Authors:** Li T. Guo, Shao L. Wang, Qing J. Wu, Xu G. Zhou, Wen Xie, You J. Zhang

**Affiliations:** ^1^Department of Plant Protection, Institute of Vegetables and Flowers, Chinese Academy of Agricultural SciencesBeijing, China; ^2^Department of Entomology, Agricultural Science Center North, University of KentuckyLexington, KY, USA

**Keywords:** *Bemisia tabaci*, nuclear DNA content, genome size, flow cytometry, k-mer analysis

## Abstract

The genome sizes of the B- and Q-types of the whitefly *Bemisia tabaci* (Gennnadius) were estimated using flow cytometry (*Drosophila melanogaster* as the DNA reference standard and propidium iodide (PI) as the fluorochrome) and k-mer analysis. For flow cytometry, the mean nuclear DNA content was 0.686 pg for B-type males, 1.392 pg for B-type females, 0.680 pg for Q-type males, and 1.306 pg for Q-type females. Based on the relationship between DNA content and genome size (1 pg DNA = 980 Mbp), the haploid genome size of *B. tabaci* ranged from 640 to 682 Mbp. For k-mer analysis, genome size of B-type by two methods were consistent highly, but the k-mer depth distribution graph of Q-type was not enough perfect and the genome size was estimated about 60 M larger than its flow cytometry result. These results corroborate previous reports of genome size based on karyotype analysis and chromosome counting. However, these estimates differ from previous flow cytometry estimates, probably because of differences in the DNA reference standard and dyeing time, which were superior in the current study. For Q-type genome size difference by two method, some discussion were also stated, and all these results represent a useful foundation for *B. tabaci* genomics research.

## Introduction

As the most fundamental genetic property of organisms, genome size refers to the amount of DNA in an un-replicated, basic, gametic chromosome set (Soltis et al., 2003). Genome size or DNA *C*-value remains a key character in biology and biodiversity, which is relevant to ecological and environmental concerns (Bennett and Leitch, 2005; Knight and Beaulieu, 2008; Greilhuber and Leitch, 2013), and is important for phylogenetic study, intergeneric classification, taxa delimitation, and hybrid identification (Zonneveld, 2001; Bures et al., 2004; Morgan-Richards et al., 2004). The accurate estimation of an organism's nuclear genome size is essential for many research questions concerning genomics, proteomics, and evolution.

Although the genome size has been estimated for more than 13,000 species of animals and plants (Bennett and Leitch, 2012; Gregory et al., 2013), genome size has been relatively understudied for invertebrates and especially for insects. Estimates of genome size can be used to guide research aimed at understanding the evolution of large-scale genomic properties of insects, and researchers have hypothesized that insect genome size relates to eusociality, parasitism, and development (Gregory, 2002; Johnston et al., 2004; Koshikawa et al., 2008). Of the nearly 1,000,000 described species of insects, genome size has been estimated for only about 793 (0.079%) (Gregory, 2015; http://www.genomesize.com). Genome size has been estimated for 1 species of Collembola, 1 species of Thysanura, 2 species of Phthiraptera, 2 species of Strepsiptera, 3 species of Mantodea, 9 species of Blattaria, 9 species of Phasmida, 14 species of Isoptera, 44 species of Orthoptera, 45 species of Hemiptera, 59 species of Lepidoptera, 113 species of Odonata, 134 species of Hymenoptera, 175 species of Diptera, and 180 species of Coleoptera. According to the animal genome size database (Gregory, 2015), the haploid 1C genome size of insects ranges from 0.09 pg for *Mayetiola destructor* to 16.93 pg for *Podisma pedestris*, with an average of 1.29 pg ± 0.10. The *C*-value has been estimated for a number of agriculturally important insect pests, including the gypsy moth, *Lymantria dispar* (Lepidoptera: Lymantriidae) at 1.03 pg, the tobacco budworm moth, *Heliothis virescens* (Lepidoptera: Noctuidae) at 0.41 pg, *Musca domestica* (Diptera: Muscidae) at 0.92 pg, the flour beetle *Tribolium castaneum* (Coleoptera: Tenebrionidae) at 0.21 pg, and the pea aphid *Acyrthosiphon pisum* (Hemiptera: Aphididae) at 0.31 pg (Gregory et al., 2013; Gregory, 2015).

Most of the data in the current genome size databases have been generated by feulgen densitometry (more recently, feulgen image analysis densitometry) or flow cytometry. These two methods have been extensively validated, and various sources of error have been identified and minimized (David et al., 2002; DeSalle et al., 2005; Hare and Johnston, 2011). However, k-mer analysis estimate based on bioinformatics method was also feasible and reasonable, recently used in many insect genome project (Wang et al., 2014; Xue et al., 2014).

Estimates of genome size have been inconsistent for the whitefly, *Bemisia tabaci* (Gennadius) (Hemiptera: Aleyrodidae), which is a severe agricultural pest (Brown and Bird, 1992; Brown et al., 1995; Oliveira et al., 2001; Brown and Czosnek, 2002). The *B. tabaci* taxon is composed of closely related sibling species, among which two members, referred to as B (Middle East - Asia Minor 1) and Q (Mediterranean), are the most invasive and destructive in many parts of the world (Delatte et al., 2009; Dinsdale et al., 2010; Xu et al., 2010; De Barro et al., 2011). The *B. tabaci* genome size has long been a subject of interest because of the status of this whitefly as a cryptic species, its haplo-diploid reproductive mode, and the genetic basis for interactions between it and its prokaryotic endosymbionts (Costa et al., 1995; Zchori-Fein and Brown, 2002). A previous estimate of the genome size of the B-type of *B. tabaci* by Brown et al. (2005) differs from our estimate, perhaps because the internal standard has been adjusted subsequent to publication of the earlier study. In this research, we used flow cytometry (*Drosophila melanogaster* as a reference standard) and combined with k-mer analysis to estimate the genome size of the B-type and Q-type of *B. tabaci*.

## Materials and methods

### Insects

The original B-type *B. tabaci* was obtained from the TH-S strain as described previously (Feng et al., 2009, 2010), and the original Q-type *B. tabaci* was collected on poinsettia (Euphorbia pulcherrima Wild. ex Klotz.) in Beijing, China in 2009. The B- and Q-types were reared on cotton plants (*Gossypium herbaceum* L. cv. Zhongmian 49) in a glasshouse under natural light at 28 ± 2°C and without exposure to chemical insecticides. The identities and purities of the B- and Q-type were confirmed by sequencing a fragment of the mitochondrial cytochrome oxidase I gene mtCOI every 2–3 months.

Heads of wild-type adult *D. melanogaster* w1118 (1C = 0.18 pg; Bennett et al., 2003) were used as the reference standard for flow cytometry measurements. *D. melanogaster* w1118 was reared in glass containers in a growth chamber (MLR-352H-PC) at 25 ± 1°C and 60 ± 5% relative humidity and with a corn culture medium as the food source.

### Sample preparation and flow cytometry

The sex of adult individuals was determined by examination with a light microscope. Each individual was placed in a separate 1.5-mL polypropylene tube. Approximately 50 female or male were treated as one replicate, and four replicates represented one sample of *B. tabaci* B or Q.

Flow cytometry protocols were as previously described (Galbraith et al., 1983, 2001; Brown et al., 2005; Doležel et al., 2007), with slight modification. Briefly, the 50 individuals in each replicate of B or Q adult female or males were collected and chopped with a razor blade in ice-cold Galbraith's buffer (pH 7.0, containing 45 mM MgCl_2_, 20 mM 3-N-morpholino propane sulphonic acid, 30 mM sodium citrate, and 0.01% (v/v) Triton X-100) in separate Petri dishes. The suspended nuclei were passed through a 40-μm-mesh filter and centrifuged at 800 g for 5 min. The supernatants were discarded, and the pellets were stained with 10 mg ml^−1^ RNase A and with 50 mg ml^−1^ PI (propidium iodide) as the fluorochrome (Johnston et al., 1999). The stained pellets, which contained nuclei, were mixed and incubated on ice in the dark for 1 h. The suspensions were then analyzed using a cell analyzer (BD LSRFortessa, BD Biosciences, New Jersey, USA) equipped with a 488-nm laser excitation source operated at an output of 100 mW. Fluorescence emission was collected with a 582/15 band-pass filter. A nucleus suspension from *D. melanogaster* w1118 adult heads was simultaneously obtained and analyzed in the same manner.

### Data analysis of flow cytometry

The raw data of nuclei peaks were processed using BD FACSDiva 7.0 software (BD Biosciences, New Jersey, USA). The nuclear DNA content of the samples was expressed as the mean ± standard error (SE). One-way ANOVAs and the Tukey test (SPSS for Windows, Rel. 17.0.0 2009; Chicago: SPSS Inc.) were used to compare the 1C genome sizes of B-type females, B-type males, Q-type females, and Q-type males of *B. tabaci*.

### Genome size estimation by k-mer analysis

To confirm our flow cytometry result, we also exacted part insert paired-end libraries (250, 500, or 800 bp; constructed in China-BGI sequencing center) sequencing data for k-mer estimation from B and Q *B. tabaci* genome project. Briefly, after optimization of k-mers, k-mer counting by SOAPdenovo (Li et al., 2010) with the k-mer size set to 17 were used, and the genome size can be estimated using the following formula: Genome size = total number of k-mers/peak value of k-mer frequency distribution.

## Result and discussion

### Result

**Figure 1:**
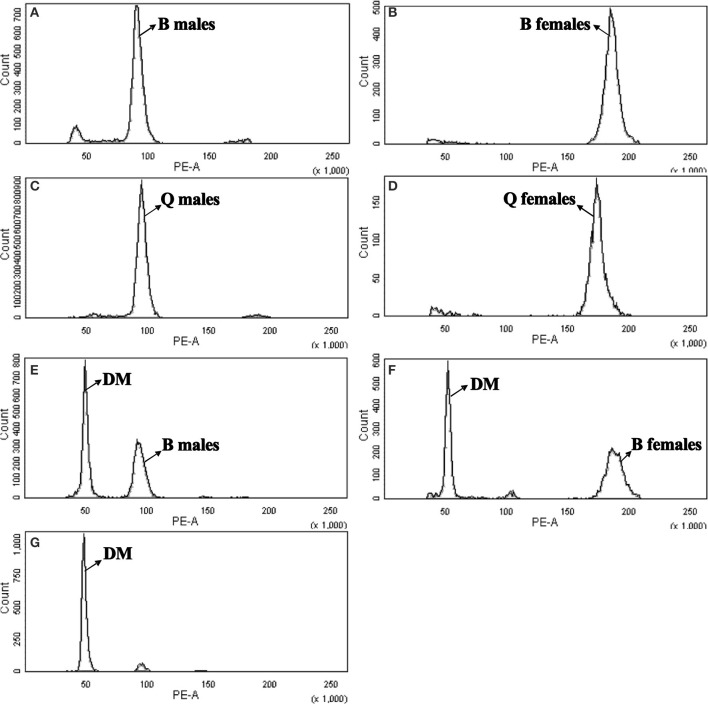
**Flow cytometry determination of the nuclear DNA content of diploid female and haploid male B-type and Q-type *B. tabaci***. *Drosophila melanogaster* was used as reference standard. The plots show the relative DNA staining of nuclei in nuclear suspensions from whole bodies (for *B. tabaci*) or heads (for *D. melanogaster*); the nuclei were stained with propidium iodide. **(A)** B-type males (1C = 0.686 pg, channel 91.37). **(B)** B-type females (2C = 1.392 pg, channel 185.455). **(C)** Q-type males (1C = 0.680 pg, channel 90.588). **(D)** Q-type females (2C = 1.307 pg, channel 174.027). **(E)** DM, *D. melanogaster* (2C = 0.36 pg, channel 48.882) and B-type males (1C = 0.686 pg, channel 93.125). **(F)** DM, *D. melanogaster* (2C = 0.36 pg, channel 51.213) and B-type females (2C = 1.315 pg, channel 187.049). **(G)** DM, *D. melanogaster* (2C = 0.36 pg, channel 47.955).

The mean nuclear DNA content of *B. tabaci* was 0.686 pg for B-type males (Figure 1A), 1.392 pg for B-type females (Figure 1B), 0.680 pg for Q-type males (Figure 1C), and 1.306 pg for Q-type females (Figure 1D); as noted in the Methods section, *D. melanogaster* head tissue cells were used as the standard, and PI was the fluorochrome. The quantity of DNA was converted to genome size according to the following relationship (Bennett et al., 2000): 1 pg DNA = 980 Mbp. The 1C genome size were estimated to be 672.2 Mbp (B-type males), 682.204 Mbp (B-type females), 666.447 Mbp (Q-type males) and 640.167 Mbp (Q-type females), respectively (Table 1). The genome size of male B-type and male Q-type differed by only 5.8 Mbp but that of females differed by 40 Mbp. The 1C genome size was smallest for Q-type females, intermediate for Q-type males and B-type males, and largest for B-type females (Table 1).

**Table 1:** **Estimates of genome sizes for males and females of the B-type and Q-type of *B. tabaci***.

**Type–sex**	**Relative fluorescence (mean ± se)**	**CV (%)**	**Nuclear DNA content (pg)**	1C Genome size (Mbp)^*^
B–male	91.370 ± 0.423	4.6–4.9	0.686 ± 0.002	672.218 bc
B–female	185.455 ± 0.827	3.4–4.1	1.392 ± 0.004	682.204 c
Q–male	90.588 ± 0.422	4.1–4.9	0.680 ± 0.005	666.465 b
Q–female	174.027 ± 1.341	3.3–3.9	1.307 ± 0.003	640.167 a

*n = 4 for each mean*.

* *Means in the column followed by different letters are significantly different (One-Way ANOVA, P < 0.05; Tukey test)*.

**Figure 2:**
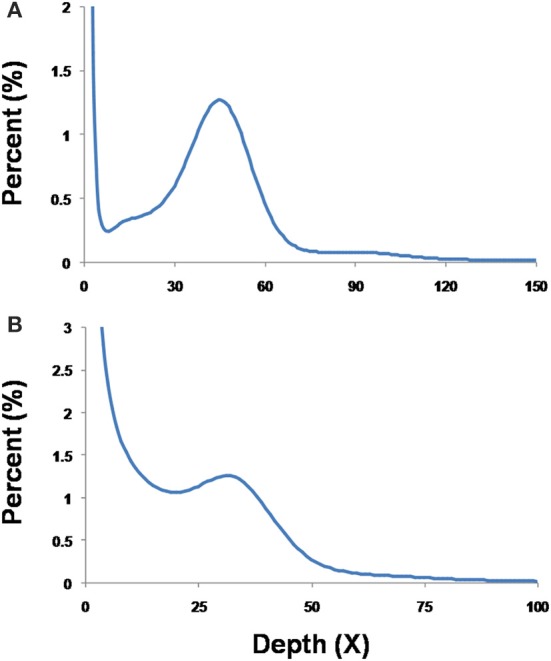
**K-mer determination of the nuclear DNA content with 17-mer frequency distribution of sequencing reads of diploid female and haploid male B-type (A) and Q-type (B) *B. tabaci***.

For B-type *B. tabaci*, the k-mer depth distributions with a minor curve (peak) at the left side shown a low level of possible heterogeneity and the genome size of B-type was estimated 681 Mb. However, for Q-type *B. tabaci*, after a series of optimization, the k-mer depth distribution graph still had not obvious normal distribution and the genome size of Q-type was estimated 720 Mb (Table S1, Figure 2).

### Discussion

The organism cell nucleus has always been the subject of intensive studies because it carries most of the hereditary material. The genome size is related with cell cycle duration, cell size and characters such as life cycle, weediness, threat of extinction and so on (Leitch and Bennett, 2007). The availability of data on genome size is critical for many fields of research, including taxonomy and evolutionary changes (Kron et al., 2007). Its knowledge is essential for gene cloning and genome sequencing projects (Rabinowicz and Bennetzen, 2006). Accurate DNA *C*-value estimates are essential for a full understanding of plant and animal genome sequencing project (Bennett et al., 2000), and further promoting function research like protein, metabolite product and physiology genomics. Our this research of accurate genome size estimation of *B. tabaci* by two methods, not only make up previous error/ incidence as erroneous estimation of genome size negatively impacts the genome sequencing project, but also strongly promote our genome-related research progress for this important pest.

To confirm our flow cytometry result, we can clearly see that genome size of B-type by two methods were consistent highly, but the k-mer depth distribution graph of Q was not enough good and the genome size of Q-type was estimated about 60 M larger than its flow cytometry result (Figure 2, Table 1). The reason for the higher Q-type genome size estimated by k-mer (even trying various k-mer parameters) was probably according to that its DNA material of constructing small libraries were not enough pure and not inbreeding like B-type *B.tabaic* (unpublished data). Based on this, Q-type genome size by k-mer estimation was on the high side and its flow cytometry result was more credible. Meanwhile, in the evaluation of nuclear DNA content of flow cytometry, choosing a suitable DNA reference standard is essential to reduce the risk of instrument nonlinearity errors and to avoid peak overlap (Doležel et al., 2007). As indicated in Figure 1, the DNA peaks including that of the DNA reference standard in the current study were discrete and unambiguous. Whether *D. melanogaster* head cells were used as an external standard (B-type male 1C = 672.218 Mbp, channel 91.37; B-type female 1C = 682.204 Mbp, channel 185.455; Figures 1A,B) or as an internal standard (B-type male 1C = 672.12 Mbp, channel 93.125; B-type female 1C = 675 Mbp, channel 187.049; Figures 1E,F), it is clear that the haploid genome size of *B. tabaci* (1C, 640~682 Mbp) is approximately 4-times larger than that of *D. melanogaster* (1C = 176 Mbp). The coefficients of variation of the DNA peaks in the current study were <5% (Table 1), indicating that the measurements were valid (Loureiro et al., 2007). In addition, *B. tabaci* males are haploid and females are diploid according to karyotype analysis and chromosome counting (Blackman and Cahill, 1998), indicating that there should be a 2-fold difference in genome size between males and females. That a 2-fold difference between the genome sizes of *B. tabaci* females and males of both biotypes was detected in our study (Table 1) provides further evidence that our estimates are valid.

In contrast to the current study, Brown et al. (2005) estimated the nuclear DNA content of male and female of the Arizona B-type *B. tabaci* to be 1.04 and 2.06 pg, respectively. We suspect that these differing results from difference in reference standard and dying time. Like Brown et al., we used PI and Galbraith's buffer to prepare the nucleus suspensions. Unlike Brown et al., we used *D. melanogaster* rather than *A. thaliana* as the DNA reference standard, and we stained nucleus suspensions with PI for 60 min rather than for 15 min. As a non-base pair-specific fluorochrome, PI can saturate in all species in 1 h, and the staining remains almost constant for 1–24 h (Bennett et al., 2003). The PI binding capacity is lower for unmethylated DNA than methylated DNA, and DNA is generally more methylated in plants (e.g., *Arabidopsis*) than in animals (Galbraith et al., 1983). We therefore suggest that differences in the DNA reference standard and dyeing time may explain the difference between the current results and those of Brown et al. (2005). We also suggest that estimates are better when *D. melanogaster* rather than *A. thaliana* is used as the DNA reference standard and when the PI staining time is 60 min rather than 15 min.

The haploid genome size of *B. tabaci* (640~682 Megabase, 20786 protein-coding genes, unpublished result) is approximately 4-times larger than that of *D. melanogaster* (176 Megabase, 17215 protein-coding genes) (Adams et al., 2000), 3-times larger than that of the honey bee, *Apis mellifera* (234.7 Megabase, 13401 protein-coding genes) (Ardila-Garcia et al., 2010), 2.5-times larger than that of the mosquito *Anopheles gambiae* (264 Megabase, 13184 protein-coding genes) (Holt et al., 2002), and 1.3-times larger that of the *Bombyx mori* (508 Megabase, 14436 protein-coding genes) (Rasch, 1974). Although *B. tabaci* does not have substantially more genes than the other insects, its genome is substantially larger. Hence, we hypothesize that a substantial part of the *B. tabaci* genome does not encode genes and contains a relatively high proportion of highly repetitive, non-coding DNA sequences.

Determining the complete genome sequence for *B. tabaci* will yield informative data and permit the hypotheses stated in the previous paragraph to be tested based on genomic and proteomic analyses. Many factors can affect genome size, such as polyploidy, fixation of accessory chromosomes, or large duplications (Uozu et al., 1997; John and Miklos, 1998; Ullmann et al., 2005); intron size (Moriyama et al., 1998); microsatellite presence (Warner and Noor, 2000); and transposable elements (SanMiguel and Bennetzen, 1998; Vieira et al., 2002). In this study of *B. tabaci*, flow cytometry measurements indicated that the genome was smaller for Q-type females than in Q-type males, and was generally smaller for Q-type adults than B-type adults regardless of sex, although the difference between B-type and Q-type males was not statistically significant. Petrov (2002) indicated that small genomes reflect high rates of DNA deletions and that these high deletion rates had over millions of years of evolution and produced quite independently of adaptation. Therefore, the differences in genome sizes of B- and Q-type *B. tabaci* documented here may reflect differences in their genomic structure. We cannot, however, make inferences about the evolutionary significance of the differences because we have estimated the genome size for only two types of *B. tabaci*. In addition, determining the specific mechanisms by which their genomes have expanded or contracted will require a closer examination of genetic characteristics such as the number of transposable elements, intron size, and number and sizes of microsatellites. Such information should help us understand the link between *B. tabaci* chromatin structure, genome size, and evolution. In summary, this study adds to the genome size database for the insect order Hemiptera. Our estimates of *B. tabaci* genome size can be used to guide whole-genome sequencing and to determine sequence integrity. The estimation of genome sizes for other *B. tabaci* cryptic species will be useful for explaining the evolutionary relationships within the *B. tabaci* species complex.

## Author contributions

Conceived and designed the experiments: LG, WX, YZ. Performed the experiments: LG. Analyzed the data: LG, WX. Contributed reagents/materials/analysis tools: LG, SW, QW, XZ, WX, YZ. Wrote the paper: LG, SW, QW, XZ, WX, YZ.

### Conflict of interest statement

The authors declare that the research was conducted in the absence of any commercial or financial relationships that could be construed as a potential conflict of interest.
