# Supplementary material for: Flow cytometry and K-mer analysis estimates of the genome sizes of Bemisia tabaci B and Q (Hemiptera: Aleyrodidae)
Source: Front Physiol. 2015 May 19;6:144. doi: 10.3389/fphys.2015.00144 (PMC4436570; doi:10.3389/fphys.2015.00144)
Supplement: Supplementary file 1 [file Table1.DOC]

**Table S1 | B and Q *B.tabaci* estimation of genome size by 17-mer distribution.**

| **Species** | **Kmer** | **Kmer num** | **Peak depth** | **Genome size (bp)** | **Used base (bp)** | **Used read** | **X** |
| --- | --- | --- | --- | --- | --- | --- | --- |
| B-type | 17 | 30651146642 | 45 | 681136592 | 37040784600 | 370407846 | 53.5714 |
| Q-type | 17 | 22733526732 | 31 | 719979235 | 27063722300 | 270637223 | 36.9048 |
